# A rare case of intravascular epithelioid hemangioendothelioma of the cephalic vein treated with surgery and postoperative radiation therapy: a case report and review of the literature

**DOI:** 10.1186/s13256-015-0565-0

**Published:** 2015-04-29

**Authors:** Maria Paola Ciliberti, Raffaella Caponio, Antonio Pascali, Gabriele Matichecchia, Marco Lioce

**Affiliations:** U.O. Radioterapia - National Cancer Research Centre - Istituto Tumori “Giovanni Paolo II”, viale Orazio Flacco, 65-70124 Bari, Italy

**Keywords:** Epithelioid hemangioendothelioma, Vascular, Intravascular, Radiotherapy, Cephalic vein, Review

## Abstract

**Introduction:**

Epithelioid hemangioendothelioma (EHE) is a rare endothelial tumor with an intermediate grade of malignancy. Few cases of primary vascular hemangioendothelioma have been described in the literature. Surgery is the treatment of choice, but radiation therapy and chemotherapy should also be considered in particular cases.

**Case presentation:**

We present the case of a 44-year-old Caucasian woman affected by EHE of the cephalic vein, treated by complete surgical removal of the mass and postoperative local radiation therapy. At 5-year follow-up, our patient is alive, with no signs of local or distant relapse and with no late radiation-related effects.

**Conclusions:**

Postoperative radiotherapy may play a role in cases in which tumor margins are close or cannot be assessed or when high-risk features are present.

## Introduction

Epithelioid hemangioendothelioma (EHE) is a rare type of endothelial tumor that demonstrates an intermediate behavior between benign hemangioma and malignant angiosarcoma. It can occur in soft tissues, bones and visceral organs but also as a primary tumor of the blood vessel. About half of EHE are primary vascular. Differential diagnosis is often difficult, but recent diagnostic tools such as immunohistochemistry and mutation research can be useful for correct characterization. Surgery is the treatment of choice, but adjuvant treatment such as radiation therapy should be considered in the case of high-risk features or when complete removal is not feasible. Although sometimes promising, chemotherapy and antiangiogenetic agents do not have a well-established role. We report the case of intravascular EHE occurring in the cephalic vein of a 44-year-old woman, treated by surgery and postoperative radiation therapy (RT).

## Case presentation

**Figure 1:**
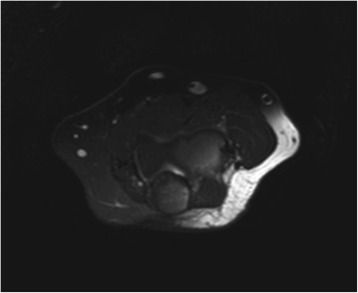
The cephalic vein is occupied by a 2×1cm mass with a weakly hyperintense T2 signal (T2W TSI SPIR image).

A 44-year-old Caucasian woman presented with a soft, painless, nonpulsatile, progressively enlarging mass at the level of the antecubital fossa of the left arm. A preliminary ultrasound (US) scan revealed an hypoechoic solid lesion along the cephalic vein, and a subsequent magnetic resonance image (MRI) of the arm confirmed a 2×1cm mass with a hypointense T1 signal and a weakly hyperintense T2 signal along the vein, suggesting a granuloma (Figure 1). Upon physical examination, the mass was mobile and painless; our patient did not present modification of reflexes of her left arm or signs of venous stasis. Our patient then underwent surgical removal of the neoplasm. Histology confirmed the diagnosis of intravenous EHE, defined as a ‘low-grade malignancy with moderate potential to recur locally or to metastatize’. Unfortunately, surgical margins were microscopically involved, so our patient underwent a second surgical operation: a 2cm segment of cephalic vein with a lozenge of skin was excised and replaced with a graft. The definitive histopathologic report confirmed the presence of microscopic residual foci of EHE, with free surgical margins. Our patient did not show any surgery-related impairment of her left arm functions. A post-surgery US study and MRI scan did not show any residual mass, while a total-body positron emission tomography-computed tomography (PET-CT) did not show any metastatic disease to regional lymph nodes or distant sites. Our patient was then referred to our radiation oncology unit. In consideration of the presence of microscopic disease foci in the second operation specimen and the considerable potential of local relapse highlighted by the pathologist, our patient underwent RT 4 months after the last surgery. A total dose of 54Gy in 27 fractions was administered with a single 6×6cm field. An 8MeV electron beam was used. Only a mild acute erythema of the irradiated skin was recorded as acute toxicity. During follow-up, our patient underwent MRI or ultrasound scans of the left arm every 6 months and computed tomography (CT) of the thorax and hepatic ultrasound scans every year. Five years after completion of the RT, our patient is alive with no evidence of recurrent or metastatic disease. She presented a Radiation Therapy Oncology Group (RTOG) grade 1 late toxicity in the irradiated skin (pigmentation changes).

## Discussion

Hemangioendothelioma (HE) is a vascular neoplasm that shows intermediate biological behavior between benign hemangiomas and highly malignant angiosarcomas. It has the potential to metastatize or recur, although less frequently than classic angiosarcoma.

In the most recent World Health Organization (WHO) Classification [1] of soft tissue sarcoma, HE is included in the group of vascular tumors of the soft tissues and of the bones. Five subtypes of HE of soft tissues are considered: Kaposiform HE (included in the intermediate-locally aggressive group), retiform HE, composite HE, pseudomyogenic HE (included in the intermediate-rarely metastasizing group) and epithelioid HE, classified as a malignant tumor.

Pseudomyogenic (epithelioid sarcoma-like) HE was the first subgroup introduced in this classification. It usually occurs in young adult men on the limbs, presenting with multiple nodules. The spindle cells are characterized by a t(7;19) translocation [2].

Kaposiform HE is a locally aggressive neoplasm that involves superficial or deep soft tissues of children and teenagers; it has features common to both capillary hemangioma and Kaposi sarcoma, with several solid poorly circumscribed nodules composed of a mixture of small capillaries and solid lobules of endothelial cells arranged in a glomeruloid pattern [3]. It is often associated with consumptive coagulopathy (Kasabach-Merritt syndrome) and lymphangiomatosis [4].

Retiform HE most often occurs in the extremities of young adults as a slowly growing single cutaneous plaque or subcutaneous nodule microscopically characterized by interconnecting arborizing blood vessels arranged in a net-like pattern and minimal cytologic atypia [5]. It has a high frequency of local recurrence but a low metastatic potential [6].

The term ‘composite HE’ indicates a locally aggressive vascular neoplasm of low-grade malignancy showing varying combinations of benign, low-grade malignant, and high-grade malignant vascular components [7].

Epithelioid hemangioendothelioma (EHE) is probably the most aggressive histological subtype. It originates from the endothelium and most often occurs in adults of both sexes, but may rarely also affect children [8]. It was first described by Weiss and Enziger [9], after a review of 14 patients, as an intermediate entity between a benign hemangioma and a high-grade angiosarcoma. The largest series of 137 cases of liver EHE was reported by Makhlouf *et al*. [10].

Histopathological evaluation is central to the diagnosis of this tumor. Biopsy and immunohistochemical assays are essential to establish a clear diagnosis and to distinguish EHE from other histological types such as carcinoma, infectious processes, metastatic adenocarcinoma such as renal cell carcinoma, Ewing’s sarcoma, telangiectatic osteosarcoma, lymphoma or multiple myeloma (plasmatocytoma), hemangioma, epithelioid hemangioendothelioma, and angiosarcoma [11,12]. Corrin *et al*. first identified the angiogenic nature of these tumors based on the identification of Weibel-Palade bodies in a kind of lung neoplasia previously called ‘intravascular bronchioloalveolar tumor’ [13].

Microscopically, EHE produces a typical dense myxochondroid or myxohyaline matrix in which endothelial cells are embedded, arranged in short strings or nests [14]. Endothelial cells are polygonal, round or occasionally fusiform. Usually, mitotic activity is infrequent and nuclei are uniform, but about one-third of EHE could present anaplastic features with a high nuclear grade, necrosis and high mitotic activity. Due to its variable histological features, differential diagnosis can be difficult. Immunohistochemistry is a useful tool: EHE shows a typical endothelial phenotype, characterized by positivity to CD31, CD34 and von Willebrand factor, and occasionally to cytokeratins [15]. Recently, some studies have proposed new markers for vascular differentiation. For example, nuclear Fli-1, a protein expressed in endothelial cells as well as in T cells and megakaryocytes, was detected in 100% of cases in a series of 13 EHE and demonstrated a better sensitivity than CD34 and better specificity than CD31 [16]. Podoplanin, a transmembrane mucoprotein expressed in lymphatic endothelium, alveolar type I cells, osteoblasts and peritoneal mesothelial cells but not in normal vascular endothelial cells, is a useful marker to distinguish EHE from nonvascular tumors [17,18]. FKBP12, which inhibits Ca2+ and calmodulin-dependent calcineurin function, regulates B and T cell responses, and is expressed in both low- and high-grade vascular tumors, is expressed in well-differentiated areas of EHE [19]. An immunohistochemical panel [20] including FKBP12 combined with CD34 and CD31 allows a 93% diagnostic sensitivity of hemangioendothelioma.

A recurrent translocation t(1;3)(p36.23;q25.1), resulting in the fusion of CAMTA1 on 1p36.23 to WWTR1 on 3q25.1, has recently been identified in most EHE, even if in different sites and grades of malignancy [21,22]; this translocation has not been detected in other pathologies like epithelioid hemangioma, epithelioid angiosarcoma or pseudomyogenic (epithelioid sarcoma-like) HE, which often mimic EHE and make diagnosis more difficult. Another recurrent oncogenic activation secondary to TFE3 gene rearrangements and common fusion with YAP1 appears to be a distinctive subset of EHE occurring in young adults, with a clinically indolent course but a high propensity to metastasis [23]. Fluorescent *in situ* hybridization (FISH) or RT-PCR analysis for these fusions may be a useful molecular diagnostic tool in challenging diagnoses.

The etiology of EHE is not well known; however, predisposing factors for angiosarcoma have been suggested to include radiation, defunctionalized arteriovenous fistula, foreign bodies, carotid endarterectomy and intravascular prosthesis [24].

After a review of 30 patients with epithelioid EHE, Mentzel *et al*. found that, although this tumor histologically has low malignancy potential, metastatic disease occurs in 20 to 30% of patients and that overall EHE carries a risk of death of up to 17%; thus, the authors suggested that it should be considered a fully malignant, rather than borderline, vascular neoplasm [25]. Local recurrence occurs in about 10 to 15% of osseous EHE cases [12] after a relatively long period of latency. Half of the metastases occur in locoregional lymph nodes or lungs [26], so periodic CT scans of regional lymph nodes and lungs are recommended in the follow-up. However, patients with metastases could be treated with surgery and then survive for a long time: only 20% of them die due to the disease after 5 years [27] because half of all metastases are in the regional lymph nodes and could be easily controlled with local surgical excision [28]. Prognosis of EHE remains better than that of classic angiosarcoma, although it often remains variable and unpredictable.

Deyrup *et al*. analyzed 49 patients with EHE in an attempt to identify a method for stratifying risk of mortality. In univariate and multivariate analysis, increasing mitotic activity and size were significantly associated with higher mortality, while tumor site, cytologic atypia, the presence of necrosis and tumor spindling were not significant. The authors concluded that large tumors (>3cm) with high mitotic activity (>3 mitotic figures per 50 high power fields) had the worst prognosis with a 5-year disease-specific survival of 59% and an increased risk of metastases (up to 25%) [8].

Clinical presentation is variable, depending on the size and location of the tumor. EHE is often asymptomatic, especially when it involves visceral organs such as the lungs or liver. Nonspecific correlated symptoms can include fatigue, anorexia, nausea, or poor tolerance to exercise [29]. When a superficial vessel is involved, EHE can present as a painful soft mass.

Radiological assessment is the first approach to identifying EHE. The imaging technique used (MRI, CT, US) depends on the primary site of the tumor. Increased uptake of 18-F-fluorodeoxyglucose (FDG) in this tumor has recently been reported [30].

EHE can affect all vascularized tissues in any site, but most frequently involves superficial or deep soft tissues, bones [31,32] and visceral organs, in particular the liver [33] and lungs [34,35]. Cases of EHE have been described in almost all sites such as the skin [36,37], central nervous system [38-40], meninges [41], lips [42], gingiva [43,44], middle ear [45], thyroid gland [46], salivary glands [47], paranasal sinuses [48,49], breast [50], pleura [51-53], lymph nodes [54], mediastinum [55,56], heart [57-61], retroperitoneum [62], ileum [63-65], peritoneum [66], testis [67], bladder [68,69], penis [70], vulva [71], and so on. In 10% of cases the disease is multifocal [72].

Mortality varies depending on primary tumor site: the mortality range is 13% for EHE of soft tissues, 31% for EHE of the bone, 43% for EHE of the liver [10] and 65% for EHE of the lung [14].

Primary vascular EHEs represent about 50% of reported cases. They arise from a blood vessel [73], commonly small- to moderate-sized veins, such as the femoral, iliac or jugular veins, but also larger vascular structures such as the aorta or vena cava. Few cases originate from medium- to small-sized peripheral vessels. The intravascular subtypes are extremely rare [74].

Usually, EHEs present clinically as a painless elastic soft mass in the vicinity of a peripheral vessel causing symptoms and signs of deep venous occlusion, ranging from edema of the extremities, weakness and ischemia, to superior vein cava syndrome. Microscopically, they are associated with a blood vessel, usually a medium-sized vein [29], showing first an expansion of the affected blood vessel and then an involvement of the surrounding soft tissues, with a centrifugal proliferation pattern. It is often difficult to make a correct diagnosis in the early phase. This is partly because they are extremely rare and also because their imaging characteristics are nonspecific, resembling inflammatory or atherosclerotic lesions [24]. Ultrasonography is useful to define the morphology and vascularization of these masses and to evaluate blood flow, usually showing intraluminal defects and altered flow patterns, loss of plasticity and velocity increase [75]. On the other hand, MRI can give additional information about the involvement of surrounding soft tissues and cleavage planes, in addition to morphological features [27]. However, definitive radiological diagnosis is often hard, and only histological diagnosis can be conclusive.

Forty-three case reports on primary vascular EHE of both veins and arteries (except the aorta) have been described in the literature (Table 1). The involved vessels were the thoracic aorta [76,77], aortoiliac segment [78], radial artery [27], inferior vena cava [79-81] , superior vena cava [82-86], innominate vein [55,87-91], azygos vein [92-94], femoral vein [73,74,78,94-98], femoral artery [99] iliac veins [28,100,101], internal carotid artery [24], axillary vein [102], brachial vein [103] or artery [104-107], popliteal artery [108], pulmonary artery [109], occipital artery [110], temporal artery [111], meningeal artery [112], and digital artery [113]. Age of presentation ranged from 11 to 79 years (mean age was 39 years), and the distribution among the two sexes was almost equal, with a slight predominance of the female sex (59% vs. 41%). The diameter of the masses was variable, ranging from 0.5cm to 11cm. Almost all patients underwent surgical removal of the vascular EHE; in four cases resection was not complete, with macroscopic residual mass or microscopic positive margins. After treatment, patients were monitored for follow-up (2 to 108 months). Six patients (14%) developed distant metastases, especially liver and lung metastases, even if this occurrence did not always affect the overall survival when metastasectomy was performed. Three patients (7%) had local relapse, often in the cases treated by incomplete surgical removal; relapse was always treated with a second surgery. Postoperative RT was administered in only five cases, and chemotherapy in three patients.

**Table 1:** **Cases of vascular epithelioid hemangioendothelioma described in literature**

**Author**	**Year**	**nr.pt**	**Sex**	**Age (yrs)**	**Artery/vein**	**Size (cm)**	**Primary therapy**	**R**	**Adjuvant therapy**	**Outcome**	**Notes**	**Follow-up (months)**
Wu *et al*. [109]	2014	1	F	58	Right pulmonary artery	/	S	0	No	NED		24
Mlynski *et al*. [96]	2013	1	F	22	Femoral vein	/	S	0	No	M	Liver and lung metastases	12
Gundara *et al.* [79]	2013	1	M	39	IVC	4.5	S	0	No	NED		48
Henton *et al.* [105]	2013	1	F	39	Brachial artery	5	S	0	No	NED		12
Muñoz *et al.* [100]	2013	1	F	23	External iliac vein	1.7	S	0	No	NED		108
Muñoz *et al.* [100]	2013	1	M	44	External iliac vein	2	S	0	RT	M	NED after liver-lung metastasectomy	96
Li *et al.* [87]	2013	1	F	38	Innominate vein	4.2	S	0	CH	NED		18
Osawa *et al.* [24]	2012	1	M	59	Internal carotid artery	8	S	2	No	LR + M	Death 6 months after surgery	6
Nutthaki *et al. *[107]	2012	1	F	42	Brachial artery	1.7	S	0	No	/		/
De Palma *et al.* [92]	2012	1	M	47	Azygos vein	1	S	0	No	NED		12
Lahon *et al.* [82]	2012	1	F	29	SVC	4,5	S	0	No	NED		10
Fulton *et al.* [94]	2011	1	F	30	Femoral vein	4	S	0	No	M	Lung metastasis at diagnosis	8
Heldenberg *et al.* [108]	2011	1	F	32	Popliteal artery	3	S	0	No	/		/
Henriquez *et al.* [80]	2011	1	M	31	IVC	7	S	2	CH	RD	Second surgery R0	/
Namaoui *et al.* [83]	2011	1	F	25	SVC	8	S	0	No	NED		6
Minyi *et al.* [73]	2011	1	F	50	Common femoral vein	3	S	0	No	NED		12
Mansour *et al.* [55]	2010	1	M	35	Innominate vein	9.5	S	0	No	NED		30
Deedar *et al.* [102]	2010	1	M	53	Axillary vein	5.8	S	0	No	NED		/
Zhang *et al.* [5]	2010	1	F	71	Brachial artery/axillary artery	6.5	S	1	No	NED		/
El Demellay *et al.* [111]	2009	1	F	41	Temporal artery	0.5	S	0	No	NED		36
Scordi-Bello *et al.* [81]	2009	1	M	35	IVC	11	S	0	No	NED		10
Tayeb *et al.* [110]	2007	1	F	29	Occipital artery	/	S	0	/	NED		12
Aydin *et al.* [99]	2006	1	M	77	Femoral artery	6	S	0	No	NED		/
Kugai* *et al.* [97]	2006	1	F	69	Common femoral vein	/	S	/	/	/		/
Castelli *et al.* [27]	2005	1	M	26	Radial artery	3	S	0	No	/		/
Ludwikoski *et al.* [101]	2005	1	F	11	Iliac vein	/	S	/	/	M	Liver metastases	18
Hampers *et al.* [113]	2002	1	F	36	Palmar arch	2.5	S	0	No	NED		12
Isowa *et al.* [88]	2002	1	F	41	Innominate vein	3.4	S	0	No	NED		28
Schröder *et al.* [98]	2001	1	F	52	Femoral vein	4	S	0	No	NED		24
Charette *et al.* [95]	2001	1	M	23	Common femoral vein	2	S	0	No	NED		18
Ferretti *et al.* [84]	1998	1	M	79	SVC	3.5	S	2	/	/	Biopsy only	/
Reix *et al.* [74]	1998	1	F	16	Common femoral vein	2	S	0	No	NED		12
Moreno *et al.* [89]	1998	1	M	33	Innominate vein	6	S	0	RT	NED	Hystiocytoid hemangioma	7
Akashi *et al.* [104]	1997	1	F	67	Brachial artery	2	S	0	No	NED		40
Zingale *et al.* [103]	1993	1	M	47	Brachial vein	/	S	0	No	LR	NED after second surgery	21
Toursarkissian *et al.* [90]	1990	1	M	62	Innominate vein	/	S	0	RT	NED		54
Delin *et al*. [78]	1990	1	F	28	Common femoral vein	/	S	0	No	NED		6
Lamovec *et al.* [93]	1990	1	F	40	Azygos vein and SVC	5	S	0	RT + CH	NED		/
Nataf *et al.* [91]	1989	1	M	48	Azygos vein	2	S	2	No	RD	Palliative S (bypass) for the presence of spinal and lymph node invasion at diagnosis	/
Harris *et al.* [28]	1989	1	M	32	External iliac vein	2,5	S	0	RT	NED		12
De Verbizier *et al*. [86]	1987	1	F	57	SVC	/	none	/	No	PD	Surgery only on liver localization	6
Yousem *et al.* [85]	1987	1	F	35	SVC	10.5	S	0	No	NED		2
Enzinger** *et al.* [9]	1982	1	/	11	External iliac vein	/	S	/	/	M		18

*Only abstract available in English language; **not available online, abstract information only. /, not specified; M, male, F, female, IVC, inferior vena cava; SVC, superior vena cava; S, surgery; RT, radiotherapy; CH, chemotherapy; NED, no evident disease; LR, local relapse; M, metastases; R, residual tumor after surgery: 0 = no residual disease, 1 = microscopic residual cells, 2 = macroscopic residual mass; PD, progressive disease; RD, residual disease; LR, local relapse.

The treatment options for EHE include surgery, RT, and chemotherapy; new therapies such as antiangiogenetics agents have been also used. Few cases of spontaneous regression of EHE have been reported [114].

When feasible, surgery with free resection margins is the treatment of choice in the management of EHE. For example, partial hepatectomy [115] or orthotopic liver transplantation [116] represent the first treatment in primary hepatic tumors. Embolization of feeding vessels causing spontaneous regression of vascular tumors has been described [117]. When feasible, a wide resection is also recommended in primary bone EHEs. A preoperative selective embolization of involved vertebra may help to reduce intraoperative blood loss [118]. Surgery is the mainstay also in the treatment of primary vascular EHE. When reconstruction of the vessel is necessary, it can be done with prosthetic or autogenous interposition graft replacement [100]. A complete local excision of the neoformation with or without local lymphadenectomy is related to long-term survival.

A few patients have been treated with chemotherapy, often in the metastatic setting, but results are not always encouraging and response of EHE to chemotherapy seems to be low. The mainstay chemotherapeutic agents for metastatic EHE are doxorubicin and fluorouracil. Intensive regimens using combinations of vincristine, doxorubicin, ifosfamide, etoposide and cyclophosphamide have shown an increase in response rate, without an advantage in overall survival [10]. Doxorubicin in the pegylated liposomal formulation seems to lead to better responses [119] and reduce the risk of cardiotoxicity [120].

Due to the endothelial origin of EHE and the good response to therapies with interferon (IFN) alpha [121,122], recent therapeutic approaches have focused on the use of antiangiogenic agents [123]. Bevacizumab is a humanized monoclonal antibody directed against vascular endothelial growth factor and seems to be an effective and well-tolerated treatment for metastatic or locally advanced angiosarcoma and epithelioid hemangioendotheliomas [124,125]. Thalidomide [126,127], lenalidomide [128], and endostar [129] are believed to have immunomodulatory and antiangiogenic properties; they also seem to be useful in the systemic treatment of EHE. Sorafenib [130] is also providing encouraging evidence of benefit.

Selective radionuclide therapy with intra-arterial injection of Yttrium (Y)-90 microspheres [131], commonly used for unresectable liver metastases and primary liver tumors with hopeful results, has shown some results in unresectable hepatic EHE.

In consideration of the high incidence of local relapse and the moderate radiosensitivity of EHE, RT has been used as adjuvant treatment in some high-risk cases, with good results. Hemangiomas involving the skeletal system have been treated effectively with local RT for many decades with a good long-term local control [132], even when RT is used as the only treatment option, such as in cases of surgically inaccessible sites [133].

Scott *et al*. [134] treated 14 patients affected by bone EHE with adjuvant or exclusive RT. The median dose administered was 54Gy for the patients treated with RT alone and 62.2Gy for the patients who underwent surgery, with a once daily (1.5 to 2Gy) or twice daily fractionation (1.2Gy). At a median follow up of 10.3 years, the 5-year local control, cause-specific survival, and overall survival rates were 100%, 86%, and 79%, respectively. No cases of late toxicity greater than G1 were recorded, suggesting that RT is a highly effective treatment option for this disease. Yin *et al*. [135] described a case of complete remission of a cervical spine EHE after the sole administration of 55Gy in 32 fractions over 43 days, using a 6MV photon three-field plan. No severe side effects were recorded, but the authors highlighted the risk of second malignancies. RT was also effective in obtaining a long-term local control when it was administered as adjuvant treatment [136], as in cases when complete surgical removal was not feasible [137,138]. No direct comparisons between surgery and radiotherapy have been published; however, high-dose radiotherapy seems to be curative when used as primary or adjuvant treatment in EHE of the spine [139,140]. Although re-resection is the treatment of choice in cases of EHE after surgery, RT showed efficacy also when administered as salvage therapy, as in the case of EHE of the mastoid described by Drazin *et al*.; the total dose administered at recurrence was 59.4Gy in 33 fractions. The patient was free from disease 8 years after surgery [48].

The role of RT in primary vascular EHE has not been established. Few data are available, and it is difficult to summarize guidelines about when this treatment should be performed, that is preoperatively, postoperatively, in the case of incomplete resection, or only at the moment of tumor progression. Hampers and Tomaino described the case of a patient with an EHE that presented as an aneurysm of the superficial palmar arch and third common digital artery [113]. The lesion was completely surgically removed and the patient received postoperative local irradiation to the hand because surgeons and pathologists could not verify tumor-free margins; dose and fraction size were not specified. The patient was free from local or distant recurrence at the 1-year follow-up evaluation. Muñoz *et al*. described a case of external iliac vein EHE with lymph node metastases treated by surgery and local RT [100]. Unfortunately, doses and volumes were not specified in the report. The patient was alive without local relapse at 8-year follow-up. Toursarkissian *et al*. described a case of EHE arising from the innominate vein treated with surgery and radiation therapy. The patient was alive at a 4.5-year follow-up without evidence of local relapse [90]. In the experience of Harris *et al*., an EHE of the external iliac vein with lymph-node involvement in a 32-year-old man was treated with surgery and adjuvant radiotherapy, because microscopic foci of the tumor were seen extending to the margins of the resection. The total dose administered was 66Gy, and the patient was free from relapse after 12 months [28].

## Conclusions

Primary vascular EHEs are rare and publications in the worldwide literature are almost exclusively case reports. Primary vascular EHEs can occur in every age group. Surgery is the preferred treatment, when feasible. Chemotherapy is often used in cases of metastatic disease, with ambiguous results. The case reported here suggests that the addition of postoperative RT may be useful in cases where tumor margins are close or cannot be assessed, or those with high-risk features. Unfortunately, EHE behavior often remains unpredictable and unrelated to microscopic findings.

## Consent

Written informed consent was obtained from the patient for publication of this case report and any accompanying images. A copy of the written consent is available for review by the Editor-in-Chief of this journal.

## Abbreviations

CT: computed tomography
EHE: epithelioid hemangioendothelioma
FDG: 18-F-fluorodeoxyglucose
FISH: fluorescent in situ hybridization
HE: hemangioendothelioma
IFN: interferon
MRI: magnetic resonance imaging
PET-CT: positron emission tomography-computed tomography
RT: radiation therapy
US: ultrasonography
